# Diffusion of active Brownian particles under quenched disorder

**DOI:** 10.1371/journal.pone.0298466

**Published:** 2024-03-04

**Authors:** Xiong-Biao Zhao, Xiao Zhang, Wei Guo

**Affiliations:** Key Laboratory of Artificial Microstructures in Yunnan Higher Education Institutions, School of Physical Science and Technology, Kunming University, Kunming, China; State University of New York at Binghamton: Binghamton University, UNITED STATES

## Abstract

The motion of a single active particle in one dimension with quenched disorder under the external force is investigated. Within the tailored parameter range, anomalous diffusion that displays weak ergodicity breaking is observed, i.e., non-ergodic subdiffusion and non-ergodic superdiffusion. This non-ergodic anomalous diffusion is analyzed through the time-dependent probability distributions of the particle’s velocities and positions. Its origin is attributed to the relative weights of the locked state (predominant in the subdiffusion state) and running state (predominant in the superdiffusion state). These results may contribute to understanding the dynamical behavior of self-propelled particles in nature and the extraordinary response of nonlinear dynamics to the externally biased force.

## 1 Introduction

The self-propelled particles, also known as active particles in modeling, can transform the internal energy into energy of motion that drives themselves out of equilibrium [1, 2] and appear in a wide variety of biological and soft matter systems. For example, at the microscopic scale, the driver protein moves on the microtubule, and swarms of bacteria [3, 4]; at the macroscopic scale, fish schools and flocks of birds [5, 6], among others. The self-propelled particles always exhibit complex interactions between them, and thus, a range of novel collective behaviors have been documented, including flocking, clustering and phase separation, and soon [7–11]. Intriguingly, even a single self-propelled particle [12–15] (without interactions) also demonstrates intricate features, such as anomalous dynamical behavior and a non-Boltzmann stationary distribution. Among various models of a single self-propelled particle [16, 17], perhaps one of the simplest is termed an active Brownian particle (ABP) in one dimension and is applied to underdamped Brownian dynamics, which is described by the Langevin equation with the inertia term. In this Langevin equation, what is crucial is not only the speed-dependent nature of the friction coefficient but also its ability to become negative over a range of velocities. The positive and negative values of the friction coefficients correspond to energy dissipation and energy absorption, respectively [18]. There are three paradigms for the speed dependent friction coefficient in one dimension [19]. First, in the Schweitzer Ebeling Tilch (SET) model, the form of the friction coefficient *γ*(*υ*) is *γ*_0_(1 − *β*/(1 + *υ*^2^)) (*β* and *γ*_0_ are two constant). If *β* > 1, the friction function *γ*(*υ*) is negative at speeds between $-\sqrt{\beta -1}<\upsilon <\sqrt{\beta -1}$ and positive at speeds either $\upsilon <-\sqrt{\beta -1}$ or $\upsilon >\sqrt{\beta -1}$ [20–24]. Second, the friction function is Rayleigh–Helmholtz (RH) friction $\gamma \left(\upsilon \right)=\gamma_{0}\left(\upsilon^{2}-\upsilon_{0}^{2}\right)$, which was originally studied by Rayleigh and Helmholtz in the propagation of sound [25]. Third, the friction coefficient derived by Schienbein and Gruler (SG) from experiments with a moving cell. The form of the friction coefficient is *γ*(*υ*) = *γ*_0_(1 − *υ*_0_/*υ*) with a discontinuity at *υ* = 0 [26]. The three friction coefficients share a common characteristic in that they either facilitate or hinder the motion of particles. This feature may lead to rich dynamic behaviors, such as anomalous diffusion and weak ergodicity. The SET model can more clearly analyze the influence of friction on the motion of active particles and the differences between ordinary Brownian particles and active Brownian particles.

Anomalous diffusion has been attracting growing interest in a wide field [24, 27–38]. Anomalous diffusion is observed experimentally, e.g., the transport of carriers in amorphous materials [27], the diffusion of particles in various ion channels on the cell membrane [28, 29], the diffusion of proteins across cell membranes [30], the active movement of mussel biological cells [31], and the movement of aphids [32]. Theoretically, anomalous diffusion has been realized through processes involving heterogeneity [33, 34], fractional Brownian motion [35], disorder systems [24, 36], and Lévy walks [37]. Anomalous diffusion deviates from normal diffusion (*α* = 1), which is conventionally defined by the power-law expansion of the particle’s mean squared displacement(*MSD*) with time, i.e., *MSD*/2*t* = (< *x*(*t*)^2^ > − < *x*(*t*) >^2^)/2*t* ∝ *t*^*α*^, where *α* represents the diffusion exponent: *α* < 0 is subdiffusive [39, 40], *α* = 0 is normal diffusion, and superdiffusive with *α* > 0 [41, 42].

The anomalous diffusion is usually accompanied by ergodicity breaking [33, 34, 39]. Ergodicity breaking can be categorized as strong and weak ergodicity breaking: i) strong ergodicity breaking indicates division of the state or phase space into mutually independent regions [43]. ii) Weak ergodicity breaking, initially introduced by Bouchaud in the context of phenomenological models of spin glass dynamics, asserted that the system’s phase space or state is not divided into mutually inaccessible regions [39, 44]. Weak ergodicity breaking has been experimentally observed in scenarios such as lipid particle diffusion in yeast cells [45], protein movement in human cell membranes [46], single-particle tracking experiments [47, 48], and tracer diffusion in mucin hydrogels [49]. Theoretically, weak ergodicity breaking has also been discovered in the scaled Brownian motion [50], the collective motion of active particles [51], and the superdiffusive transport arises in generic Hamiltonian systems [52].

Extensive exploration has taken place over recent decades regarding the diffusive transport of a single particle within potential landscapes [24, 53]. Potential landscapes can be divided into spatiotemporal randomness [54, 55], spatial periodicity [53], or spatial randomness [24, 56]. The term “quenched disorder” typically refers to spatially random potential without temporal dependence. Practically, the surfaces of most solids are not composed of perfect crystals, often featuring random inhomogeneities, including natural genetic material [57]. Theoretically, this randomness can be modeled by space-dependent disorder potential [56].

It is generally assumed that the overdamped Langevin equation provides a quantitative description of the dynamics of a classical Brownian particle in the long time limit [58]. Another research avenue involves examining active systems with substantially reduced damping, allowing inertial effects to become significant. It is important to note that a basic difference between macrobiotic and microbiotic is their Reynolds number (Re), which characterizes the ratio of inertial to viscous forces. Micro-scale organisms like bacteria predominantly experience swimming at extremely low Reynolds numbers, where damp forces predominate, their motion is frequently characterized as overdamped dynamics. Experimentally, including trapped microspheres, two-level systems, single molecules are described by an overdamped Langevin equation [59]. At the macroscopic scale, such as fish swimming, which occurs at high Reynolds numbers and is dominated by inertial forces, the case of underdamped dynamics is considered. In the presence of inertia, numerous nonlinear systems can manifest various collective dynamic behaviors, including solitons, nonlinear waves, and shock phenomena [60].

Previous studies have demonstrated non-ergodic anomalous diffusion in underdamped Brownian dynamics with quenched disorder [61–64], anomalous diffusion and transport in the SET model with a periodic potential [20], and diffusion of active particles subjected to additive and multiplicative noise [65]. The investigation into the diffusion of active Brownian particles has primarily centered on elucidating the impact of noise, often neglecting the consideration of the energy landscape in the environment—particularly the space-dependent random potential. Additionally, the intentional introduction of external forces plays a pivotal role in perturbing the symmetry of the system [18, 38, 66, 67]. Building upon this foundation, we investigate anomalous diffusion and weak ergodicity breaking in the SET model with quenched disorder, considering the external force *F* and the characteristic friction coefficient *β*. By regulating the two parameters of the external force *F* and the characteristic friction coefficient *β*, non-ergodic subdiffusion, non-ergodic superdiffusion, and non-ergodic normal diffusion are observed. These anomalous diffusion and weak ergodicity breaking predominantly stem from the relative weights of the locked state (dominant in the subdiffusion state) and the running state (prevalent in the superdiffusion state).

The rest of the paper is organized as follows. In section 2, the dynamic equation (the Langevin equation) is given. In section 3, some measurement definitions are introduced. In section 4, numerical simulation results on anomalous diffusion and ergodicity breaking are presented and discussed. In section 5, the conclusion of the article summarizes the results.

## 2 Model

We consider the motion of an underdamped active Brownian particle driven by an external force in a Gaussian random potential. The motion of each particle is described by the dimensionless Langevin equation (*LE*) [24]:

$$\begin{array}{c} \dot{x}=\upsilon ,\dot{\upsilon }=-\gamma \left(\upsilon \right)\upsilon -V^{\prime }\left(x\right)+F+\sqrt{2D(\upsilon )}\eta \left(t\right), \end{array}$$ (1)

where *x* is the position of the particle at time *t*. The dot and prime denote derivatives with respect to *t* and *x*, respectively. *η*(*t*) is Gaussian white noise, whose mean is zero, 〈*η*(*t*)〉 = 0, and the correlation function 〈*η*(*t*)*η*(*t* + *τ*)〉 = *δ*(*τ*)(〈…〉 denotes statistical averages). *F* is the external force. *γ*(*υ*) is the friction coefficient in the form of the SET model, and *D*(*υ*) is the noise intensity, satisfying the fluctuation-dissipation relation

$$\begin{array}{c} \gamma \left(\upsilon \right)=\gamma_{0}\left(1-\frac{\beta }{1+\upsilon^{2}}\right),D\left(\upsilon \right)=\left|\gamma \left(\upsilon \right)k_{B}T\right|, \end{array}$$ (2)

where *γ*_0_ is the original friction coefficient. *k*_*B*_ denotes the Boltzmann constant. *T* is the temperature. *β* is the characteristic factor of the friction coefficient. If *β* > 1, $-\sqrt{\beta -1}<\upsilon <\sqrt{\beta -1}$, the friction coefficient *γ*(*υ*) is negative. The frictional force drives the motion of particles. Outside this range, $\upsilon <-\sqrt{\beta -1}$ or $\upsilon >\sqrt{\beta -1}$, the friction coefficient *γ*(*υ*) is positive. The frictional force hinders the motion of particles. As $\upsilon =\pm \sqrt{\beta -1}$, *γ*(*υ*) = 0 means friction disappears. If *β* ≥ 1, *γ*(*υ*) ≥ 0, the frictional force is a damping force that hinders the motion of particles.

The quenched disorder *V*(*x*) is a particular family of random potentials that obeys a zero mean and Gaussian correlation function

$$\begin{array}{c} \left\langle V\left(x\right)V\left(x^{\prime }\right)\right\rangle =\sigma^{2}e^{\left(-\frac{|x-x^{\prime }{|}^{2}}{2\lambda^{2}}\right)} \end{array}$$ (3)

where *σ* is the dispersion of the potential fluctuations, and *λ* is the characteristic correlation length of the quenched disorder.

## 3 Observables

The ensemble-averaged mean squared displacement (*MSD*) for a diffusion process follows a power law of time

$$\begin{array}{c} \left[\left\langle x^{2}\left(t\right)\right\rangle -{\left\langle x\left(t\right)\right\rangle }^{2}\right]/2t\propto t^{\alpha }. \end{array}$$ (4)

The diffusion exponent *α* < 0 and *α* > 0 correspond to the subdiffusion and the superdiffusion of the system, respectively [68]. Compared with the *MSD*, many experiments based on a single particle tracking are evaluated in terms of the time averaged *MSD* (*TMSD*) [34, 39]:

$$\begin{array}{c} \bar{\delta^{2}\left(t\right)}=\frac{1}{t_{w}-t}\int_{0}^{t_{w}-t}{\left[x\left(t^{\prime }+t\right)-x\left(t^{\prime }\right)\right]}^{2}dt^{\prime }, \end{array}$$ (5)

where *t*_*w*_ is the length of the time series and *t* is the lag time. *TMSD* changes randomly in certain circumstances. In order to more accurately study the ergodicity of the system using the single particle trajectory, the time- and ensemble-averaged *MSD* (*TEMSD*) was introduced, i.e., [34, 69],

$$\begin{array}{c} \langle \bar{\delta^{2}\left(t\right)}\rangle =\frac{1}{t_{w}-t}\int_{0}^{t_{w}-t}\langle {\left[x\left(t^{\prime }+t\right)-x\left(t\right)\right]}^{2}\rangle dt^{\prime }. \end{array}$$ (6)

Moreover, the ergodicity breaking parameter (*EB*) is used to quantitatively analyze the weak ergodicity breaking of the system [70, 71]

$$\begin{array}{c} EB=\frac{\langle {\bar{\delta^{2}\left(t\right)}}^{2}\rangle -\langle \bar{\delta^{2}\left(t\right)}\rangle^{2}}{{\left\langle \bar{\delta^{2}\left(t\right)}\right\rangle }^{2}}. \end{array}$$ (7)

It can effectively describe the fluctuation change of the time-averaged mean square displacement. In stationary processes, *EB* = 0 is a sufficient condition for ergodicity. Besides, the amplitude scatter distribution *ϕ*(*ξ*) describes the dispersion and distribution of the *TMSDs* around *TEMSD*, which is also used to check ergodicity. The dimensionless parameter *ξ* is defined by

$$\begin{array}{c} \xi =\frac{\bar{\delta^{2}\left(t\right)}}{\left\langle \bar{\delta^{2}\left(t\right)}\right\rangle }. \end{array}$$ (8)

The amplitude scatter is a Gaussian distribution centered at *ξ* = 1. It implies that the system exhibits ergodicity [72].

## 4 Results and discussions

Numerical simulations of Eq (1) use the second-order Runge–Kutta method. In most cases, 25 particles over 80 different realizations of the potential are simulated, i.e., the statistical ensemble averages over 2000 particles. The particles are initially uniformly distributed along a space of length 5000λ, and the initial velocity follows the Maxwell distribution at temperature *T*. Thus, these particles experience diffusion and independent parts of the potential to avoid statistical correlations. The potential landscape covers *N* = 2^26^ lattice points with a lattice constant of *δx* = 0.2. Other parameter values are *T* = 0.1, time step Δ*t* = 0.01, and disorder correlation length λ = 1. Numerical simulation results for observables are shown below.

**Fig 1:**
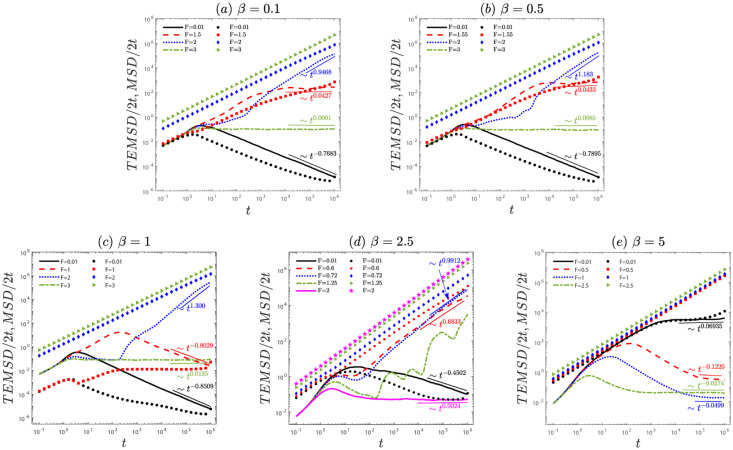
The anomalous diffusion of an underdamped active Brownian particle governed by Eq (1), for different values of the external bias *F* with (a) *β* = 0.1, (b) *β* = 0.5, (c) *β* = 1, (d) *β* = 2.5, and (e) *β* = 5. The ensemble mean squared displacement (*MSD*/2*t*, marked by lines) with Eq (4) and the time- and ensemble-averaged mean squared displacement (*TEMSD*/2*t*, marked by symbols) with Eq (6). The remaining parameters are: $\gamma_{0}=1,\sigma =\frac{\sqrt{2}}{2},\lambda =1,N=2^{26},\Delta x=0.2,T=0.1$, and the length of the time series *t* = 10^6^. The representative dynamic properties are indicated by the (dotted) fitted lines.

The anomalous diffusion is investigated by the *MSD*/2*t* in Fig 1. For the small and moderate values of the characteristic factor *β* (e.g., *β* = 0.1, 0.5, 1 and 2.5, see (Fig 1a–1d)), the fitted exponent *α* increases from *α* < 0 to *α* > 0 and then decreases to approach zero (in the time scale *t* ∼ [10^5^, 10^6^]) with an increase in the external force *F*. This implies that particle diffusion transitions from subdiffusion to superdiffusion and finally to normal diffusion as the value of the external force increases. Physically, in the case of weak force, quenched disorder plays a predominant role in the system, a large number of particles cannot pass through the potential barrier, i.e., the locked state (subdiffusion). As the force increases, the influence of quenched disorder on the system gradually weakens, a large number of particles can pass through the potential barrier, i.e., the running state (superdiffusion). In the case of strong force, quenched disorder becomes unimportant and particles follow the force direction and thus normal diffusion appears. Note that in Fig 1(d) (*β* = 2.5), uncertain diffusion is observed in the vicinity of *F* = 1.25. However, for a large value of the characteristic factor (e.g., *β* = 5, see Fig 1(e)), the system exhibits normal diffusion(*α* ≈ 0) for different external force. To sum up, as the external force or the characteristic factor increases to a larger value, the system transitions from anomalous diffusion to normal diffusion in Fig 1. Note that discrepancies between the *MSD*/2*t* and *TEMSD*/2*t* are observed (see Figs 4(a) and 5(a)). It indicates the potential occurrence of ergodicity breaking in the system.

**Fig 2:**
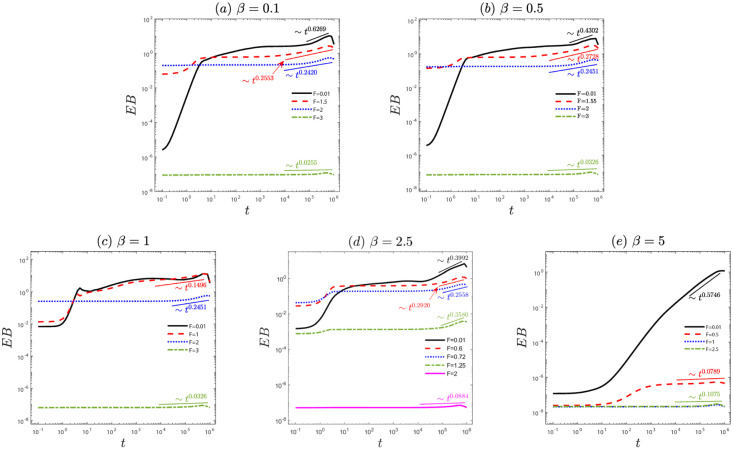
The ergodicity breaking parameter *EB* corresponds to Fig 1: (a) *β* = 0.1, (b) *β* = 0.5, (c) *β* = 1, (d) *β* = 2.5, and (e) *β* = 5, for diffusion values of the external bias *F*. The remaining parameters are the same as Fig 1.

**Fig 3:**
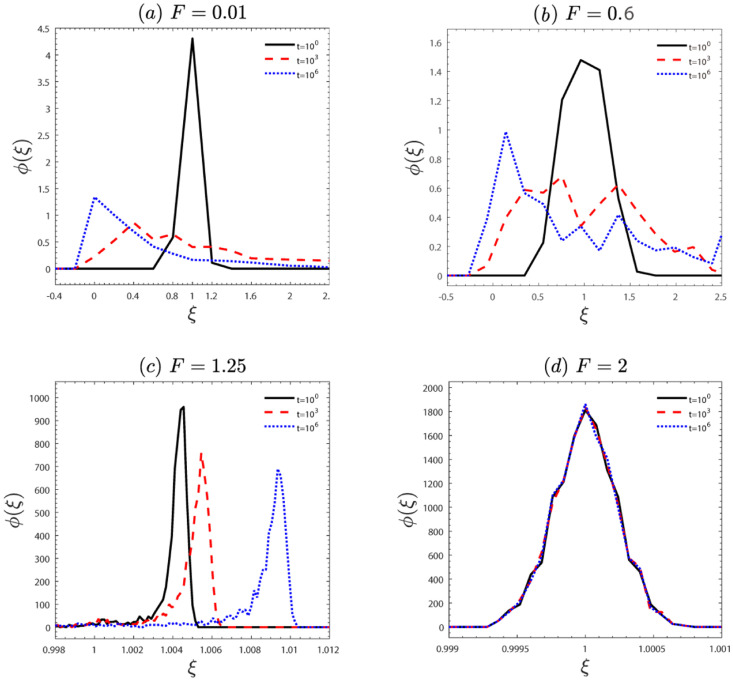
The amplitude scatter distribution *ϕ*(*ξ*). The (*a*-*d*) corresponds to *β* = 2.5 with (a) *F* = 0.01, (b) *F* = 0.6, (c) *F* = 1.25, (d) *F* = 2. The other parameters are the same as in Fig 1.

We analyze the ergodicity of the system through the ergodicity breaking parameter and the amplitude scatter distribution in Figs 2 and 3. For small and moderate external force *F*, the ergodicity breaking parameter *EB* ≠ 0 (see Fig 2). It means that the ergodicity of the system is break. For the large external force *F*, the *EB* approaches zero, i.e., the system is ergodicity. Furthermore, the ergodicity can be further confirmed by the amplitude scatter distribution in Fig 3a–3d. For lag time *t* ∼ 10^6^, the distribution of *ξ* has distinct peaks at *ξ* ≠ 1 in Fig 3a–3c. It demonstrates that the ergodicity is broken. However, Fig 3(d) displays an amplitude scatter that follows a Gaussian distribution centered at *ξ* = 1, signifying system ergodicity. These results provide further confirmation that the system recovers ergodicity as the external force increases.

**Fig 4:**
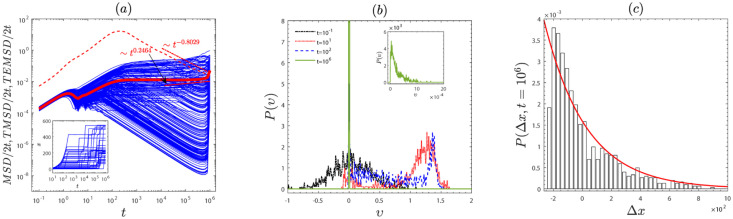
The subdiffusion case with the emblematic parameter values *F* = 1 and *β* = 1. (a) The *MSD*/(2*t*), *TMSDs*/(2*t*) and *TEMSD*/(2*t*) are indicated by the dotted line, thin and thick solid lines, respectively. The inset in (a) shows the evolution of the trajectories at the time *t* = 1 × 10^6^. (b) The velocity distribution at typical times *t* = 1 × 10^−1^, 1 × 10^1^, 1 × 10^2^, 1 × 10^6^. The inset in (b) is an enlarged view of the velocity distribution at the time *t* = 1×10^6^. (c) The distribution of particle positions *P*(Δ*x*) (Histogram) at the time *t* = 1 × 10^6^, here Δ*x* = *x* − 〈*x*〉. The distribution of position is fitted by the exponential function (red line): *P*(Δ*x*) = *a* exp(−*b*Δ*x*) with *a* = 0.001585 and *b* = 0.00346. The remaining parameters are the same as in Fig 1.

**Fig 5:**
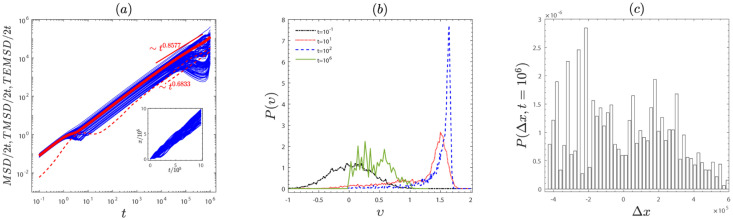
The superdiffusion case with the emblematic parameter value *f* = 0.6 and *β* = 2.5. (a) *MSD*/(2*t*), *TMSD*/(2*t*) and *TEMSD*/(2*t*) are marked by the dotted lines, thin and thick solid lines, respectively. The inset shows the evolution of the particle’s trajectory over time. (b) The velocity distribution at typical times *t* = 1 × 10^−1^, 1 × 10^1^, 1 × 10^2^, 1 × 10^6^. (c) the distribution of particle positions *P*(Δ*x*) (Histogram) at the time *t* = 1 × 10^6^, where Δ*x* = *x* − 〈*x*〉. The remaining parameters are the same as in Fig 1.

To gain insights into the non-ergodic subdiffusion and the non-ergodic superdiffusion, the *TMSDs*, the trajectories, the velocity distributions, and the position distributions of the particles are presented in Figs 4 and 5. *TMSD*/2*t* is randomly scattered around *TEMSD*/2*t* in Figs 4(a) and 5(a). It demonstrate that the ergodicity of the system is broken. For the subdiffusion case, the particle’s trajectories burst intermittently in the direction of movement (see the inset in Fig 4(a)). For the subdiffusion case, the position of the particle varies approach linearly with time (see the inset in Fig 5(a)), since the frictional force acts as the driving force that, together with the external force, provides energy to particles.

Moreover, Figs 4(b) and 5(b) show the velocity distributions for the subdiffusion case and the superdiffusion. For the subdiffusion case (see Fig 4(b)), a second peak emerges near the running state *υ* = *F*/*γ*(*υ*) (*υ* ≈ 1.5 for *F* = 1 and *γ*(*υ*) = 0.68) at *t* = 10^2^, as certain particles are influenced by the external force. But over time, the second peak disappears gradually due to the frictional force becoming a damping force that impedes the motion of the particles. Eventually, the particles are trapped in a few of the potential wells, going into the locked state (*υ* = 0). For the superdiffusion case (see Fig 5(b)), the peak appears of velocity distribution near the running state *υ* = *F*/*γ*(*υ*) (*υ* ≈ 1.7 for *F* = 0.6, *γ*(*υ*) = 0.36) at *t* = 10^2^. But as time increases, this peak gradually approaches the locked state *υ* = 0. The reason is that $\upsilon >\sqrt{\beta -1}\approx 1.2$ (*β* = 2.5), and the friction coefficient *γ*(*υ*) > 0, friction acts as a damping force that prevents particles from moving in a positive direction. As time increases, the speed gradually decreases until it is less than $\sqrt{\beta -1}$ (*β* = 2.5), the friction force acts as the driving force, providing energy for the motion of the particles. Besides, the position distributions are non-Gaussian distributions in Figs 4(c) and 5(c). It also indicates that the system is anomalous diffusion.

Eventually, we will make some comments on the observed phenomena. For the small and moderate values of *β*, non-ergodic anomalous diffusion, including non-ergodic superdiffusion and non-ergodic subdiffusion, is observed (see Figs 1a–1d and 2a–2d). For the large value of *β*, the system is normal diffusion (Figs 1(e) and 2(e)) [73]. Besides, for the small and moderate external force case, as *β* increases, the proportion of *β*/(1 + *υ*^2^) in the friction coefficient increases gradually. The friction force changes from damping force to driving force, and the diffusion changes from non-ergodic superdiffusion to non-ergodic normal diffusion. In the case of large *F*, the effects of noise, potential, and friction are overshadowed, and particle movement is primarily driven by *F*, the system is ergodic normal diffusion.

## 5 Conclusions

We have reported a study on the anomalous diffusion and ergodicity breaking of an active particle with the speed-dependent friction coefficient driven by a constant external force on quenched disorder. First, we observed anomalous diffusion (Fig 1) and the system’s ergodicity (Figs 2 and 3). In Fig 1a–1d, with an increase in the external force *F*, particle diffusion transitions from subdiffusion to superdiffusion and finally to normal diffusion. In Fig 2, as the characteristic factor *β* increases, for small external forces *F*, indicating a break in the system’s ergodicity, for the moderate external force *F*, signifying the restoration of system ergodicity, for the large external force *F*, the system is ergodic. Note that for the same characteristic factor *β*, increasing the external force *F* leads to the recovery of system ergodicity. Physically, this is because for the large external force, the potential becomes negligible, causing the particle to align with the force direction (homogenization), resulting in the ergodic process. Furthermore, the ergodicity also can be further confirmed by the amplitude scatter distribution in Fig 3. To gain deeper insights into the non-ergodic subdiffusion and non-ergodic superdiffusion (in Figs 4(a) and 5(a)) under weak and moderate external force *F* or characteristic factor of friction coefficient *β*. This behavior can be attributed to the relative weights of the locked state (predominant in the subdiffusion state) and running state (predominant in the superdiffusion state). However, the large external force *F* or characteristic factor of friction coefficient *β* contributes to the system restoring ergodic normal diffusion. Physically, the external force *F* drives the particle into the running state from the locked state in the velocity space during potential barrier crossing. And the characteristic friction coefficient factor *β* can modulate the self-propulsion behavior of the particles, facilitating their transition into the running state. These findings may contribute to understanding the dynamical behavior of self-propelling particles in nature and the extraordinary response of nonlinear dynamics to external bias force.
